# A consensus exercise to inform the future of National Health Service General Dental Services using a modified-Delphi technique

**DOI:** 10.1038/s41415-025-9100-x

**Published:** 2026-04-10

**Authors:** Ivor G. Chestnutt, Rachael Pattinson, Paul Brocklehurst, Anwen Louise Cope

**Affiliations:** 41415549736001 Dental Public Health, Cardiff University School of Dentistry Heath Park, Cardiff, UK; 41415549736002 Public Health Wales, Cardiff, UK

## Abstract

**Background** Problems relating to the commissioning and delivery of National Health Service (NHS) General Dental Services (GDS) have been well-documented.

**Objectives** This study aimed to gain consensus on issues deemed of importance to the future delivery of dental care in NHS GDS.

**Methodology** Consensus was gained using a modified-Delphi technique. This method asks respondents the degree to which they agree or disagree with the statements on a nine-point Likert scale. Consensus is achieved when more than 70% of respondents either agreed or conversely disagreed with the statement.

**Results** General dental practitioners (n = 70) agreed on 24 of 33 statements. There was consensus on statements relating to a more preventively orientated service and it was possible to identify agreement on what might constitute a core dental service such as the concept of a ‘shortened dental arch' and the exclusion of molar endodontics from NHS GDS. However, the idea that NHS dental care should be provided only to children and a defined subset of the population, with for example, those earning over a predefined threshold, ineligible for NHS dental care failed to achieve consensus.

There were no significant differences between dentists' views relating either to age or NHS contractor status (performer/provider) when it came to agreement/disagreement on the issues scrutinised.

**Conclusions** This work provides pointers as to the current views of dentists in Wales and could inform future development of NHS GDS.

## Key points

- This work provides a contemporary view of what general dental practitioners believe should be offered via the NHS General Dental Service.
- The work should inform the future development of the commissioning and delivery of NHS primary dental care.
- The work shows that whilst a consensus could be achieved on what constitutes a core dental service, there was not overall agreement on the restriction of such a service to a defined subset of the population.

## Introduction

The issues that have impacted the effective and efficient delivery of National Health Service (NHS) General Dental Services (GDS) have been debated and recorded in much detail in recent years.^1^^,^^2^^,^^3^^,^^4^^,^^5^^,^^6^^,^^7^ These are summarised in Box 1. The inadequacy of the 2006 contract used by NHS commissioners to contract with independent general dental practitioners for the provision of the NHS GDS in England and Wales was apparent from shortly after its inception.^8^^,^^9^^,^^10^ Whilst its shortcomings have been widely acknowledged, some 18 years later, satisfactory contract reform is still an on-going aspiration rather than a realised objective.

Evolution rather than revolution has been the approach taken by the department of health in London and Welsh Government in Cardiff, but progress has been slow. Two things are clear:

1. There is not enough money to provide a comprehensive state-funded dental service in England and Wales, has not been since 1952, and there never will be
2. Our politicians are reluctant to state this as a fact.

Despite much reporting of the problems facing the GDS, there has been little investigation of what a future NHS GDS should entail. From a professional perspective, what do experienced general dental practitioners want and expect? Should the bullet be bitten and state funded care restricted only to the vulnerable? Should NHS care be limited to a core service? These are amongst the issues that were investigated in this work.

Box 1 A summary of issues currently affecting the delivery of NHS General Dental Services in Wales

- Current demand for NHS General Dental Services is outstripping supply
- The current contracting mechanism is not conducive to managing high-needs patients and has been generally regarded as not fit for purpose
- Dentists are seeing fewer patients than was the case pre-COVID pandemic
- There are issues with recruitment of dentists, particularly in rural and remote areas
- The cost-of-living crisis is affecting patient ability to afford dental care
- A period of high inflation after a long period of low inflation is impacting on dental practices and their costs
- An increasing proportion of dentists are either opting out of providing NHS dentistry or are reducing their dental contract
- Dentistry has not embraced the use of skill-mix in the way that has occurred in healthcare more generally.

## Aims

The study had three aims:

- To gain consensus on questions deemed of importance to the future delivery of dental care in NHS GDS
- To determine if there are differences in consensus between two groups of practitioners: i) those who hold NHS GDS contracts; and ii) those dentists who do not directly hold NHS contracts
- To examine if views vary dependant on time since qualification.

## Methodology

### Study design

#### The Delphi and modified Delphi processes

This study was conducted using a modified Delphi technique. This research process has been in existence since the 1950s and was developed by the Research and Development Corporation for technological forecasting.^11^^,^^12^ The technique is designed to achieve agreement on issues by a group of ‘experts' in possession of either explicit or implicit knowledge.

The ‘experts' (study participants) are asked to rate their agreement with a given statement via an anonymised questionnaire survey. The results of the survey are analysed and statements on which there is agreement are removed from the questionnaire. In a second round the statements on which consensus is not achieved are circulated again, but on this occasion the participants are given feedback on the level of agreement in the previous round and offered the opportunity to change their score.

**Fig. 1:**
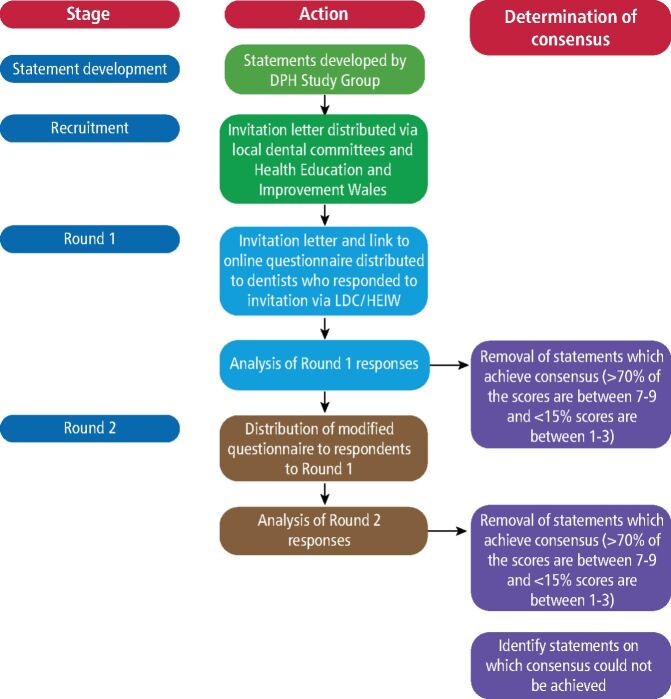
Study procedures

In this study, a modified-Delphi technique was employed, in which consensus was sought on a set of pre-generated statements.^13^ These statements, developed by the All-Wales Dental Public Health Study Group, were identified as important for the future delivery of dental care in Wales. The study design is illustrated in Figure 1 and followed standard modified-Delphi methodology.^11^^,^^12^^,^^14^ Non-respondents received a reminder to complete the questionnaire seven days after the initial mailing.

### Study participants and their recruitment

#### Eligible for inclusion

The study participants whose consensus was sought were general dental practitioners and dental trainees selected from two pools: i) members of local dental committees (LDCs) who were invited to participate via the LDC secretary, supplemented by a snowballing technique via LDC members;^15^ and ii) dentists who were participating in foundation and core training via Health Education and Improvement Wales (HEIW).

#### Not eligible for inclusion

- Members of the dental team who are not dentists
- Members of the public or patients.

### Questionnaire

In line with the modified Delphi technique,^13^ the items on which consensus was sought were generated *a priori*. The items generated by the All-Wales Dental Public Health study group were peer reviewed and agreed a thematic of relevance to the future development of the NHS GDS and provision of dental care in Wales.

The questionnaire comprised 33 statements on which consensus was determined. An open question provided respondents the opportunity to add additional comments. Finally, five questions sought demographic data which asked about the background and experience of those responding.

#### Questionnaire software

The study employed the Jisc^16^ online survey tool under a Cardiff University license. This is compliant with general data protection requirements and is overseen by the university's information technology department.

#### Dissemination of the questionnaire

Potential participants were emailed via the LDCs and HEIW in the first instance and invited to participate in the study. Those interested in participating were invited to contact the study team. From there, they were sent a personalised link to the questionnaire.

#### Delphi rounds

Whilst provision was made for three rounds of questionnaire distribution, the degree of consensus gained after two rounds was deemed unlikely to be changed significantly in a third round. The study was therefore limited to two rounds.

#### Number of participants

There is no agreed formula for determining the ideal number of participants in a Delphi study.^13^ Previous work has had as few as eight participants and others into the thousands. For this exercise, 70 participants were recruited for round one. It is accepted that a sample of this size may not be reflective of the wider population of general dentists' views but is within the range of participants typically used in a Delphi study.

#### Data collection, analysis and definition of consensus

Participants were asked to rate their strength of agreement with the given statements on a nine-point Likert scale. Consensus on agreement was defined as >70% of the scores for a given item are between 7–9 and <15% scores are between 1–3, on the 1–9 Likert scale. Consensus on disagreement was defined as >70% of the scores for a given item are between 1–3 and <15% scores are between 7–9, on the 1–9 Likert scale.

#### Quantitative analysis

For each item, the proportion of participants scoring each of the nine response options per statement was calculated. The response options where then trichotomised (completely disagree, strongly disagree, disagree/mildly disagree, neither agree nor disagree, mildly agree/agree, strongly agree, completely agree). Consensus on agreement and disagreement as defined above was then determined.

Statements on which consensus was achieved during the first round of the exercise were removed and the remaining statements were circulated for a second time together with the average scores recorded at the first time of asking.

Differences between contract and non-contract holders were examined using chi-square test (proportion of respondents) agreeing with a given statement between rounds. The differences in response scores by those qualified for 20 years or less were compared with those qualified for 21 years or more. The level of significance was set at 0.05.

#### Qualitative analysis

Responses to the open question were grouped by theme and sub-themes.^17^

#### Research ethics

Research ethics review and approval for this work was provided by Cardiff University Dental School Research Ethics Committee (Ref. DSREC 24/05). Participants consented to participate by responding to an email invitation and indicating their consent when completing the online survey.

## Results

### Study participants

In total, 70 dentists participated in the study. All responded to the first round of the study and 67 (96%) responded to the second round.

The demographic characteristics of the respondents are described in Table 1. Respondents were equally distributed between those who owned (51%) and those who did not own (49%) a dental practice. Similarly, just under half (48%) of all respondents held an NHS general dental service contract, with 38% acting as a performer/associate and not holding an NHS contract.

**Table 1:** Demographic characteristics of the dentists who participated in the study

**Characteristic**	**Round 1 (% respondents) (n = 70)**	**Round 2 (% respondents) (n = 67)**
**Ownership of a dental practice**		
Yes	51	54
No	49	46
**Capacity in which NHS care is provided**		
I am a provider/principal and hold an NHS dental contract	48	48
I am a performer/associate and do not hold an NHS dental contract	38	37
I currently work in a trainee position within NHS dentistry	6	4
Other	9	10
**Hours per week providing direct NHS clinical care (excludes time spent on administration)**		
I do not provide NHS GDS clinical care	11	12
More than 0 but less than 5 hours	10	10
6–10 hours	7	6
11–15 hours	9	10
16–20 hours	10	6
21–25 hours	10	18
26–30 hours	13	15
31–35 hours	19	13
36–40 hours	10	9
More than 41 hours	1	0
**Number of years qualified as a dentist**		
0–5 years	6	4
6–10 years	14	13
11-15 years	11	12
16–20 years	23	19
21–25 years	20	22
26–30 years	10	12
31–35 years	4	7
36–40 years	7	4
More than 41 years	4	4
GDS, General Dental Services; NHS, National Health Service		

A total of 43% of performers spent 26 hours or more per week providing direct NHS clinical care, with 11% not themselves providing NHS GDS care. The modal time since qualification was between 16 and 20 years. Additionally, 54% of respondents had been qualified for 20 years or less.

### Consensus agreements

#### Consensus after the first round

Of the 33 statements on which participants were asked to give an opinion, consensus was achieved on 13 (ten in agreement and three in disagreement) after the first round. That left 20 statements on which no consensus was achieved. Of these 19 were distributed in the second round. A question on increasing NHS patient charges in Wales to bring them more in line with those in England was removed as changes made to charges in Wales in April 2024 made that statement redundant.

#### Consensus after the second round

After the second round, consensus was achieved on a further eleven statements (nine agree, two disagree), leaving eight statements on which no consensus was achieved.

Those statements on which consensus was achieved are shown in Table 2. Those which failed to reach the threshold for consensus after two rounds are shown in Table 3.

**Table 2:** Statements on which consensus was achieved, majority agreeing

**Consensus, majority agreeing**
Tooth whitening should not be available on the NHS Patients are responsible for their own oral health Dental care professionals should be allowed to offer oral health advice to patients NHS patients should be charged for non-attendance Leaving aside the value of the fee, a fee per item system of remuneration is preferable to the current banding system Patients presenting for an urgent appointment should pay a higher charge than when attending for a routine scheduled check-up Patients should pay in advance of their planned appointments for NHS dental care The application of fluoride varnish to all adult patients irrespective of dental caries-risk is an inefficient use of NHS resources Patients with moderate or severe dental pain that is not relieved by over-the-counter analgesics should be seen by a dental professional within 24 hours A ‘core' NHS dental service should offer only intra-coronal restorations, extractions and plastic dentures There should be a greater emphasis on directing children from the Designed to Smile programme to general dental practices for ongoing care Adult patients who are at low risk of dental disease (indicated by three green scores on the assessment of clinical oral risks and needs tool) should not be entitled to a dental recall examination more frequently than once every twelve months NHS Wales should seek to enhance the availability of Level 2 providers, i.e., dentists with enhanced skills at sub-specialist level NHS Wales should accept the concept of a ‘shortened dental arch', i.e., retention of anterior and premolar teeth as a priority Molar endodontics should not be provided by the NHS The focus on NHS dental care should be on prevention of disease Health Boards should keep a centralised waiting list for patients wanting to access NHS general dental services. Practices should be able to claim a did not attend fee from the NHS when patients fail to attend appointments without due notice A greater degree of mixing NHS and private care should be possible than the regulations currently allow, e.g., patients should be allowed to make a ‘top-up' payment to enhance the aesthetic qualities of a crown provided via the NHS.
**Consensus, majority disagreeing**
There should be greater use of remote consultations in NHS dental care No mixing of NHS and private dental care should be allowed. Patients should be treated either totally privately or totally via the NHS Dental therapists should be able to hold an NHS dental contract independent of a dental practitioner, i.e., they should be able to contract directly with health boards Patients should be allowed the flexibility to select their own recall intervals Patients with moderate or severe dental pain that is not relieved by over-the-counter analgesics should expect to wait up to three days for ‘face-to-face' care.
NHS, National Health Service

**Table 3:** Statements on which consensus was not achieved

**No consensus, majority agreeing**
As a condition of training in a UK dental school, newly qualified dentists should be required to undertake a period of working in the NHS general dental service (beyond foundation training) The concept of dental registration for NHS patients should be reintroduced Some argue that the concept of universal provision of NHS dental care in Wales is unaffordable. Therefore, NHS care should be provided only to children and a defined subset of the population, with for example, those earning over a predefined threshold, ineligible for NHS dental care Urgent NHS dental care should be arranged through dedicated urgent dental care centres.
**No consensus, majority disagreeing**
Associates (performers) should be able to hold their portion of the NHS contract independently and be accountable for underperformance against that contract Patients who are non-compliant with oral hygiene instructions should be discharged from the practice.
**No consensus, majority neither agreeing nor disagreeing**
Weighted capitation (i.e., a regular payment per-patient with the amount paid based on their likely dental need) is the best method of remunerating dental practitioners ‘Ring-fenced' appointments, proportionate to the size of a practice's NHS contract, should be kept available for patients seeking urgent NHS dental care.
NHS, National Health Service

### Views on the envisaged future of the NHS dental service

#### Universal coverage

Consensus was not achieved in response to the suggestion that as universal provision of NHS dental care in Wales is unaffordable it should be restricted to children and a defined subset of the population – only 57% agreed with this position.

#### Prevention

There was consensus that NHS dental care should focus on prevention (73%) and near universal agreement (93%) that patients are responsible for their own oral health. However, consensus was not achieved on the suggestion that patients who were non-compliant with oral hygiene instructions should be discharged from the practice.

There was consensus that universal application of fluoride varnish to adult patients was an inefficient use of resources. Greater emphasis on directing children from the Designed to Smile^18^ oral health improvement programme to general dental practices for ongoing care was agreed (78%) as was the role of dental care professionals in offering oral health advice to patients (93%).

#### Treatment services

There was a consensus that NHS Wales should accept the concept of a shortened dental arch, that is, the retention of anterior and premolar teeth as a priority (74%). Consensus was also achieved that molar endodontics should not be provided by the NHS (74%). Respondents agreed that a ‘core' NHS dental service should offer only intra-coronal restorations, extractions and plastic dentures (78%). There was very high agreement (93%) that, as at present, tooth whitening should not be available via NHS general dental services.

#### Managing urgent care

Patients in moderate or severe pain that could not be relieved by ‘over the counter' analgesics, should be seen by a dental professional with 24 hours (82%). The suggestion that such patients could wait for up to three days was rejected (73%). There was no consensus on the idea that urgent care should be arranged through dedicated urgent dental care centres. Similarly, there was no agreement that ‘ring-fenced' appointments proportionate to the size of a practice's NHS contract be kept available for patients seeking urgent dental care.

Consensus was achieved (71%) that health boards should keep a centralised waiting list of patients wanting to access NHS GDS.

#### Recalled attendance

Regarding the frequency of recalled attendance, there was consensus that adult patients at low risk of dental disease should not be entitled to a recall appointment more frequently than once every 12 months (75%). There was marked disagreement with the suggestion that patients should be allowed to select their own recall intervals (77%).

#### Funding NHS dental care

There was consensus (86%) that a fee-per-item method of remuneration was preferable to the unit of dental activity banding system. Weighted capitation as the best method of remunerating dental practitioners did not achieve consensus. Similarly, those in favour off the reintroduction of patient registration did not reach the 70% threshold required for consensus – 62% being in favour of this way of organising patient relationships with a dental practice.

Other forms of funding for NHS services such as sessional payments or personal dental service contracts were not included in the survey.

It was agreed that payment in advance of their planned appointments for NHS dental care (84%) and that patients attending an urgent appointment should pay more than that due for a scheduled attendance (85%).

There was substantial support (89%) for charging patients who failed to attend an appointment, and 73% were in favour of being able to claim a fee from the NHS when patients fail to attend.

There was consensus (71%) that a greater degree of ‘mixing', i.e., joint funding where patients were able to supplement NHS provided care by making ‘top-up' payments. The example given related to patients being allowed to make an additional payment to enhance the aesthetics of a standard crown provided by the NHS. In total, 88% rejected the view that patients should be treated either totally via the NHS or privately.

#### Skill-mix and contacting arrangements

Views differed on skill-mix, dependant on the scenario asked about. There was support (74%) for Level 2 providers, that is, NHS Wales should seek to enhance the availability of dentists with sub-specialist level skills. However, there was strong consensus that dental therapists should not be able to hold a dental contract directly with a health board, independent of a dental practitioner. Whilst not achieving consensus a majority of respondents (66%) disagreed that associates (performers) should be able to hold their portion of the NHS contract independently and be accountable for underperformance against the contract.

#### Expectations of newly qualified dentists

There was no consensus that as a condition of training in a UK dental school, newly qualified dentists should be required to undertake a period of work in the NHS general dental services beyond foundation training.

#### Remote consultations

There was strong consensus against (90%) the greater use of remote consultations in NHS dental care.

### Differences of views by characteristics of the respondents

Chi-square analysis was conducted to determine differences in views of the respondents in relation to two variables, time since qualification and whether they held an NHS dental contract (as a provider rather than a performer). Relevant statements thought likely to elicit different responses according to these variables were examined. However, no significant differences were observed. As an example, no significant differences were observed between those holding NHS contracts and those not holding such contracts when asked whether, ‘associates (performers) should be able to hold their portion of the NHS contract independently and be accountable for underperformance against that contract' (Table 4).

**Table 4:** Comparison of views between those holding an NHS dental contract (provider) and those not holding an NHS dental contract (performers) on whether performers should be able to hold their portion of the NHS contract independently and be accountable for underperformance against that contract

**Statement**		**Capacity in which care is provided**	
		**Provider** **N (%)**	**Performer or other** **N (%)**
Associates (performers) should be able to hold their portion of the NHS contract independently and be accountable for underperformance against the contract	Disagree	18 (26.1)	12 (17.4)
	Neither agree nor disagree	10 (14.5)	20 (29.0)
	Agree	5 (7.2)	4 (5.8)
	**Total**^†^	**33 (47.8)**	**36 (52.2)**
Differences not statistically significantly different. ^†^Data for one respondent missing. NHS, National Health Service.			

## Discussion

### Who should qualify for NHS dental care?

There is currently insufficient resource (both money and clinicians) to provide universal state funded dental care on a population basis. However, there is unlikely to be substantial new injections of cash into the general dental service. On a local basis however, a lack of funding may not be the major issue. Rather, flaws in the current contract make it insufficiently attractive to both attract and retain practitioners and to deliver care for high needs patients.

In this exercise, no consensus was reached as to whether NHS dental care should therefore be provided to only children and a defined subset of the population. It seems logical, however, that if dentists are to be paid a greater fee for looking after high-needs patients, then fewer patients can be seen within a defined and non-increasing cash ceiling.

The question therefore remains, in the absence of significant (i.e., doubling) of investment into NHS dentistry, should the NHS seek to formally agree who is and who is not entitled to NHS dental care based on need or ability to pay (i.e., explicit rationing of services)? The alternative is the current default approach where entitlement to care is supposedly universal but in reality, is down to where you live, the length of local waiting lists and ability to navigate the system. This is another form of rationing but is inherently implicit in nature.

### Preventive dental care

A long-time ethos of reforming NHS dental care has been the need for greater emphasis on prevention, and this was generally accepted by respondents, although there was a consensus that the universal application of certain preventive treatments may be inefficient. This would indicate support within the GDS for targeted prevention. For example, directing children from the targeted school- and nursery-based national oral health programme, Designed to Smile, to general practice was favourably received and this is an element of the programme that could be progressed.

The Steele Report^19^ on the future of general dental services, included a model where only those patients with a stable oral environment, where disease risks are managed and when the patient is established in a continuing care relationship should be entitled to more advanced restorative procedures. There was not, however, a consensus that patients failing to comply with oral hygiene instruction be removed from a dental practice, which seems reasonable and equitable since this may be influenced by factors outside the patient's control.

### What care should be provided?

Whilst there was no consensus on restricting to whom NHS dental care should be provided, consensus was more readily reached on restriction of the items of care that should be provided under NHS GDS (‘clinical rationing'). Respondents reached consensus on the concept of a shortened dental arch and the removal of molar endodontics from state-funded care. These, plus agreement on what a ‘core' service should include, may provide commissioners with ideas on how limited resources may be used to limit what can be provided.

### The provision of urgent dental care

At a time when access to dental care is problematic, this exercise has provided a useful insight into how practitioners think urgent (emergency) dental care should be organised. There was agreement on how quickly urgent care should be delivered. However, recent initiatives or suggestions that patients be directed to urgent dental care centres or that practices should be required to reserve appointments for emergency dental care in relation to the magnitude of their NHS contract were not supported. There was consensus that a greater fee should be payable for an urgent than a scheduled appointment. While this approach may be seen to favour regular attenders, those most likely to attend in pain are those who are least likely able to afford dental care.

### Recalled attendance

Related to regular attendance, the frequency of recalled attendance is of relevance. In Wales low risk patients as judged by green scores on the assessment of clinical oral risks and needs toolkit^20^ are not entitled to attend for a ‘check-up' more frequently than once every 12 months and this was supported. Disagreement with patients selecting their own recall intervals is also an important finding, supportive of demand management.

### Funding mechanisms

The future arrangements for securing NHS GDS relate to how dentists are paid and the fundamentals of different contracting mechanisms. There was strong consensus that fee per item was viewed as preferable to the unit of dental activity method of contracting, but weighted capitation, whereby providers are reimbursed a fixed amount per patient, dependent on need, failed to reach consensus.

When the current contracting arrangements were introduced in 2006, charging for failure to attend a scheduled appointment was prohibited, much to the displeasure of many practitioners. There was strong consensus for the reintroduction of charges for patients who fail to attend their appointment. This may of course, exacerbate oral health inequalities. Also reaching a consensus view was that the NHS should be liable to pay a fee to the dentist in these circumstances, an unlikely aspiration.

### Skill-mix

It has been argued that failure to adopt a skill-mix approach has hindered effective delivery of dental care,^21^ and that protectionism on the part of dentists towards the greater use of dental therapists and dental hygienists has limited their potential. Other factors such as scope of practice and earning differentials between dental hygienists and dental therapists may also have influenced the great adoption of skill-mix. In the present work, while there was agreement on the expansion and greater use of Level 2 providers, there was strong consensus that dental therapists should not be able to contract directly with health boards or hold their own contract. Similarly, a majority were against associates (performers) holding and being responsible for underperformance on their portion of a contract.

Regarding younger dentists, views have been expressed that there should be a ‘tie-in' to NHS dentistry. NHS England have consulted on this issue recently, saying it did not apply to medicine but did to dentistry because of the cost of training dentists. However, as far as the Welsh dentists taking part in this exercise are concerned, consensus was not achieved that a period of work in the NHS should be mandated in addition to foundation training.

Finally, there was strong disagreement on the greater use of remote consulting. During the Coronavirus pandemic there was much interest and suggested scope for the greater use of teledentistry. This work suggests that that is not an avenue for which there is any degree of enthusiasm amongst most dentists in Wales.

### Patient and public perspective

While the results of this study are relevant to the delivery of dental care and hence to members of the public and dental patients, in this exercise we were solely seeking the views of dentists. We therefore expressly did not involve a patient public involvement representative in this work. The questions on which consensus was being sought are technical and consensus desired from dentists' perspective in this instance.

## Conclusions

This work demonstrates support amongst respondents for the NHS GDS. It highlights areas where there is likely to be support for changes to how dental services are contracted for and delivered, but also area on which there is no fixed view on the issues enquired after.

## Data Availability

Data are available from the corresponding author upon reasonable request.
